# Supplementary figures and images for: Ribosome binding protein GCN1 regulates the cell cycle and cell proliferation and is essential for the embryonic development of mice
Source: PLoS Genet. 2020 Apr 23;16(4):e1008693. doi: 10.1371/journal.pgen.1008693 (PMC7179835; doi:10.1371/journal.pgen.1008693)

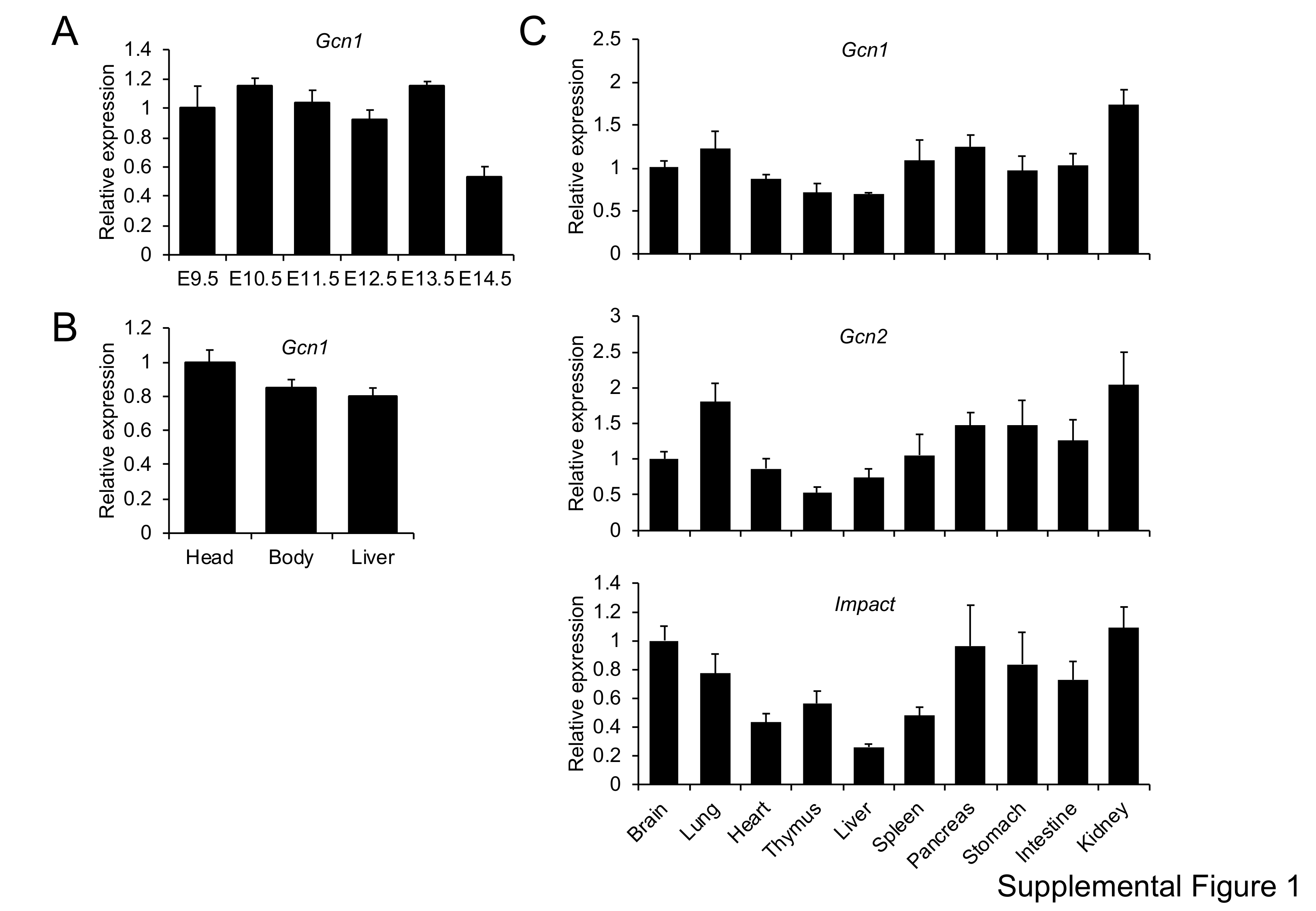

Supplement: S1 Fig — (A) Gcn1 expression in WT embryos at each stage was quantified by RT-PCR. The values for E9.5 were set to 1, and the results are shown as relative means±SD from multiple independent animals (N = 4). (B) Gcn1 expression in each part of the WT embryos at E14.5 was quantified by RT-PCR. The value for the head was set to 1, and the results are shown as relative means±SD from multiple independent animals (E9.5-E14.5: N = 4). (C) Gcn1, Gcn2, Impact expression of each organ of the WT embryos at E18.5 was quantified by RT-PCR. The value for the brain was set to 1, and the results are shown as relative means±SD from multiple independent animals (N = 3). (TIF) [file pgen.1008693.s001.tif]

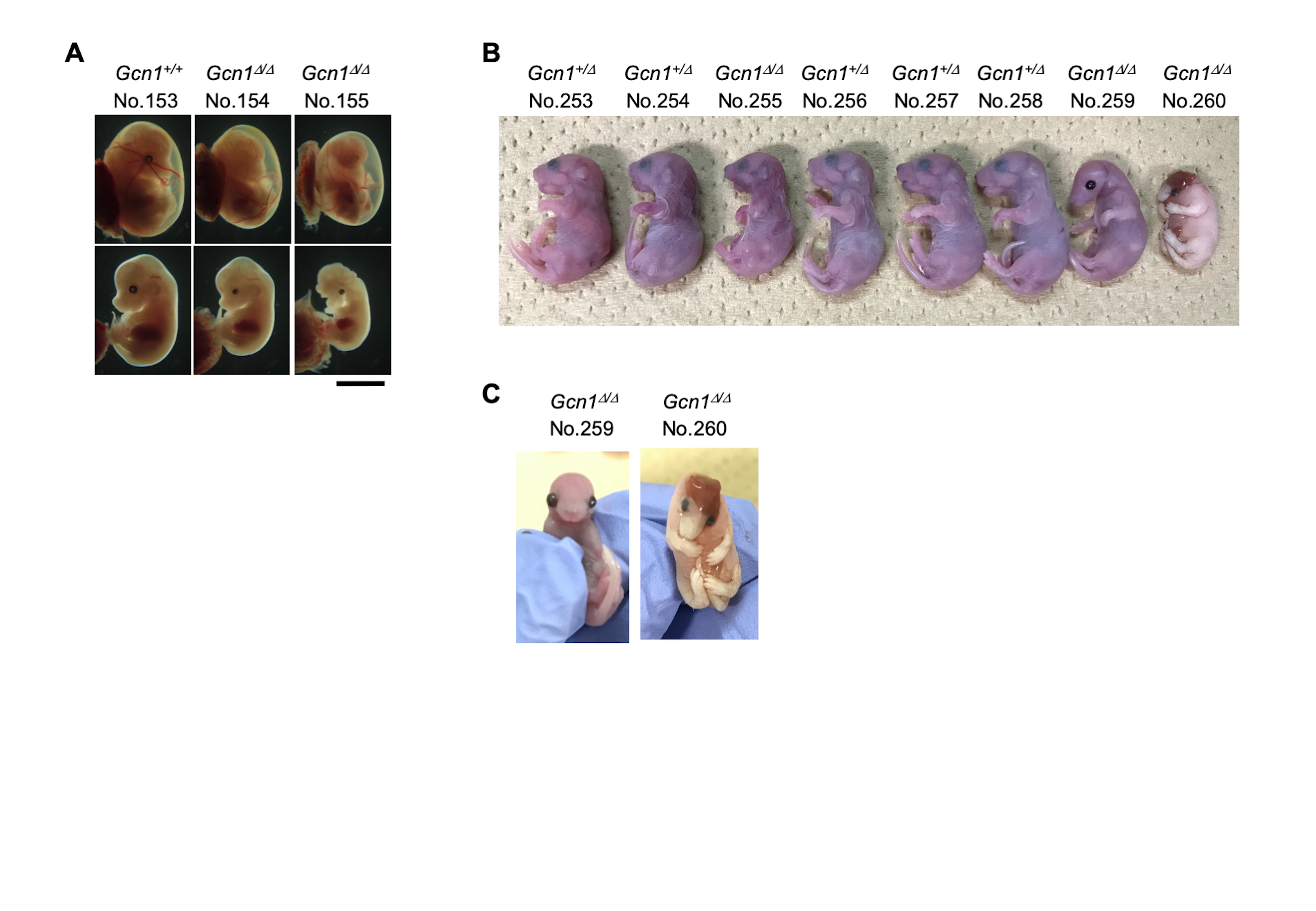

Supplement: S2 Fig — (A) Representative pictures of the embryos at E14.5. Scale bar: 5 mm. No.155 of Gcn1ΔRWDBD embryo showed abnormalities in the head or had an anencephaly-like phenotype. (B) Representative pictures of the embryos at E20.5. No.260 of Gcn1ΔRWDBD embryo was dead at the time of cesarean section and showed abnormalities in the head or had an anencephaly-like phenotype. (C) Pictures of face of Gcn1ΔRWDBD embryo at E20.5 in S2B Fig. No.259 and 260 of Gcn1ΔRWDBD embryos showed mandibular hypoplasia and exophthalmos/hypoplasia of the eyelid. (TIFF) [file pgen.1008693.s002.tiff]

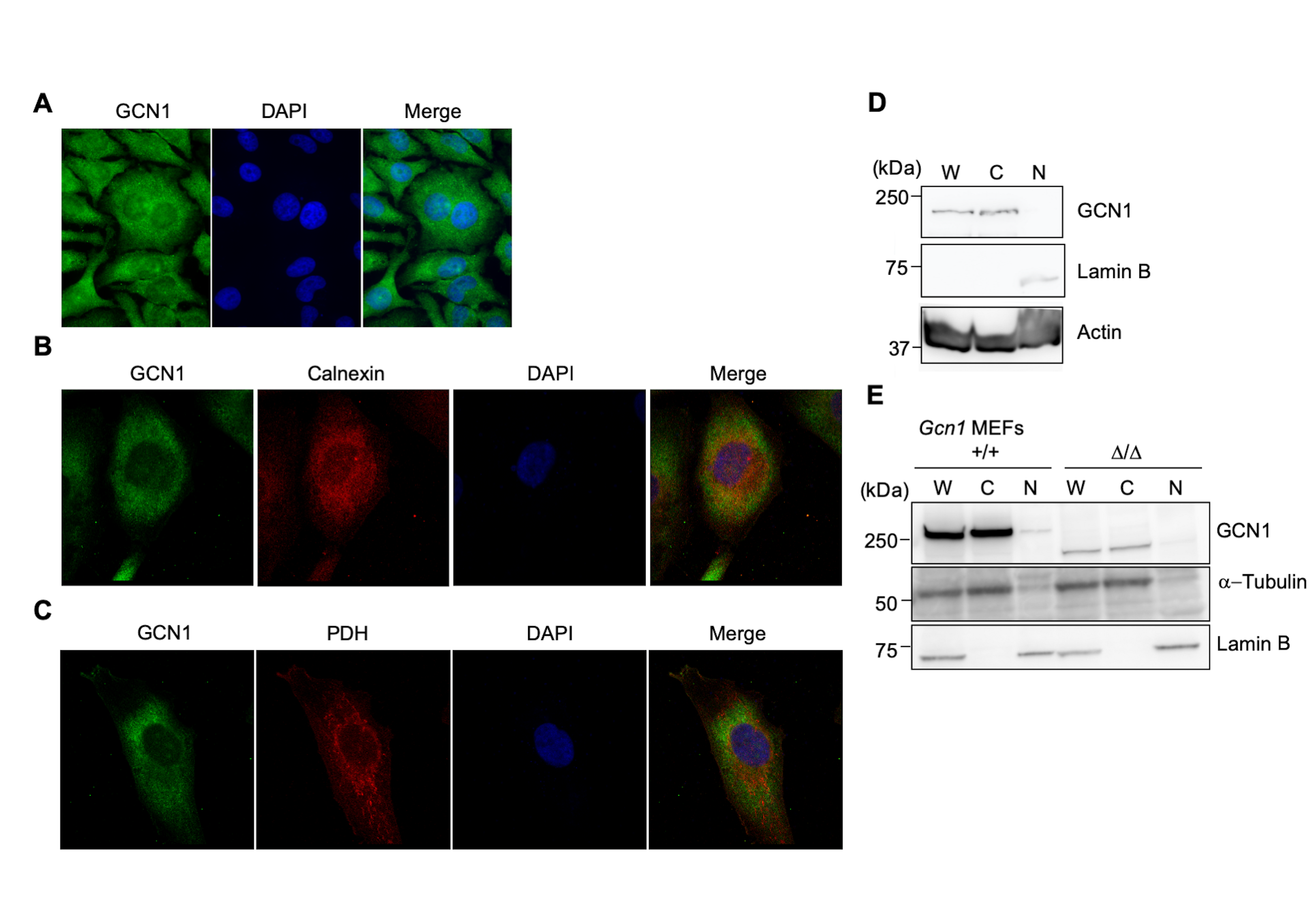

Supplement: S3 Fig — (A) Immunofluorescence analysis of GCN1 in HeLa cells. GCN1 localization is shown in green, and nuclear DAPI staining is shown in blue. The merged images are also shown. (B)(C) Double immunofluorescence staining of GCN1 (green) and calnexin (red) (B) or PDH (red) (C) in HeLa cells. Nuclear DAPI staining is shown (blue). The merged images are also shown. (D) HeLa cells were fractionated into cytosol (C), nuclear (N) and whole cell (W) fractions and subjected to immunoblot analysis to detect GCN1, Lamin B and β-actin. (E) MEFs were fractionated into cytosol (C), nuclear (N) and whole cell (W) fractions and subjected to immunoblot analysis to detect GCN1, α-Tubulin and Lamin B. Equal amounts of proteins were subjected to SDS-PAGE. (TIFF) [file pgen.1008693.s003.tiff]

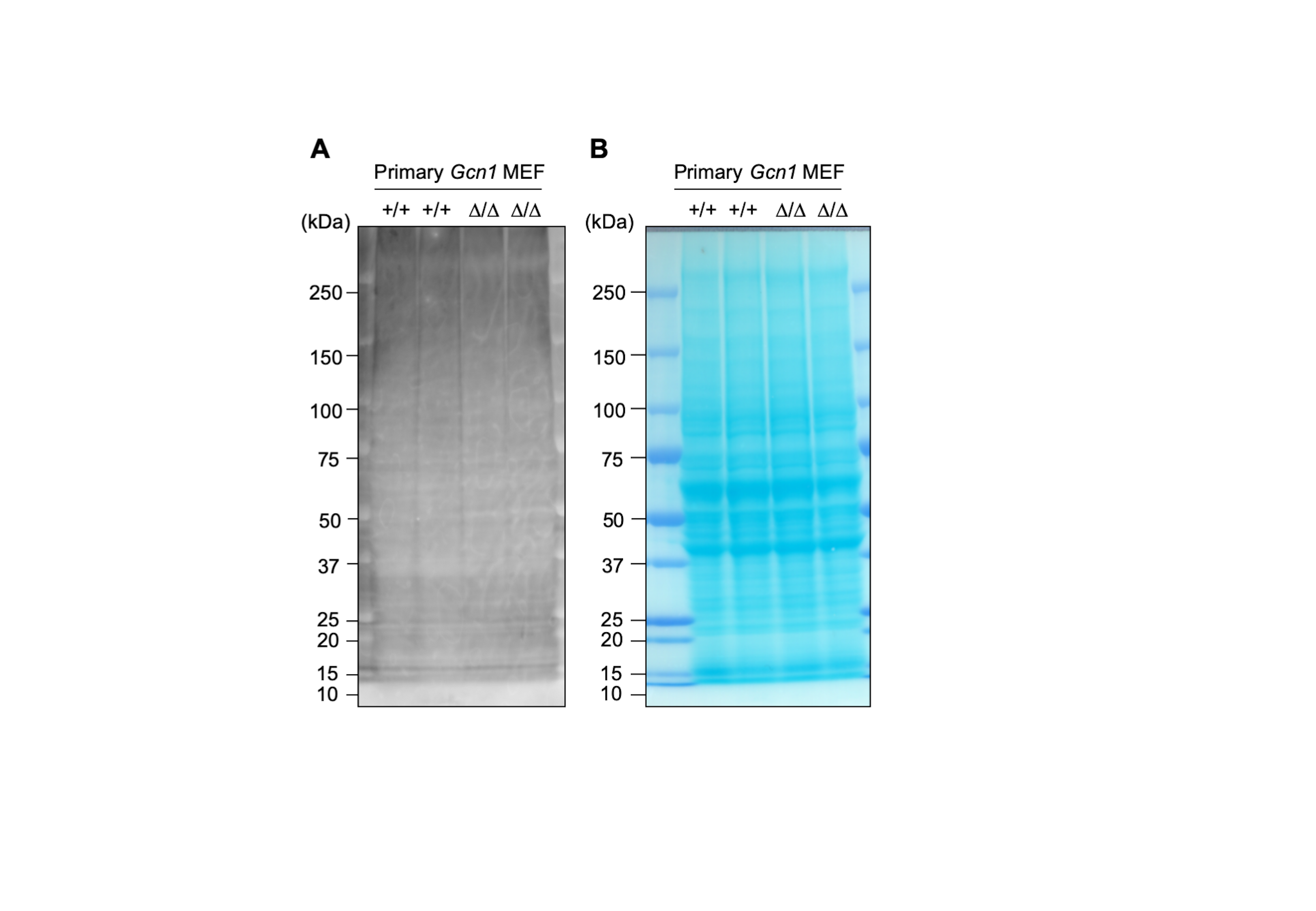

Supplement: S4 Fig — (A) De novo synthesized proteins in the Gcn1+/+ and Gcn1ΔRWDBD MEFs were measured using L-azidohomoalanine (AHA). (B) Protein levels were also confirmed by protein staining on the same membrane. (TIFF) [file pgen.1008693.s004.tiff]

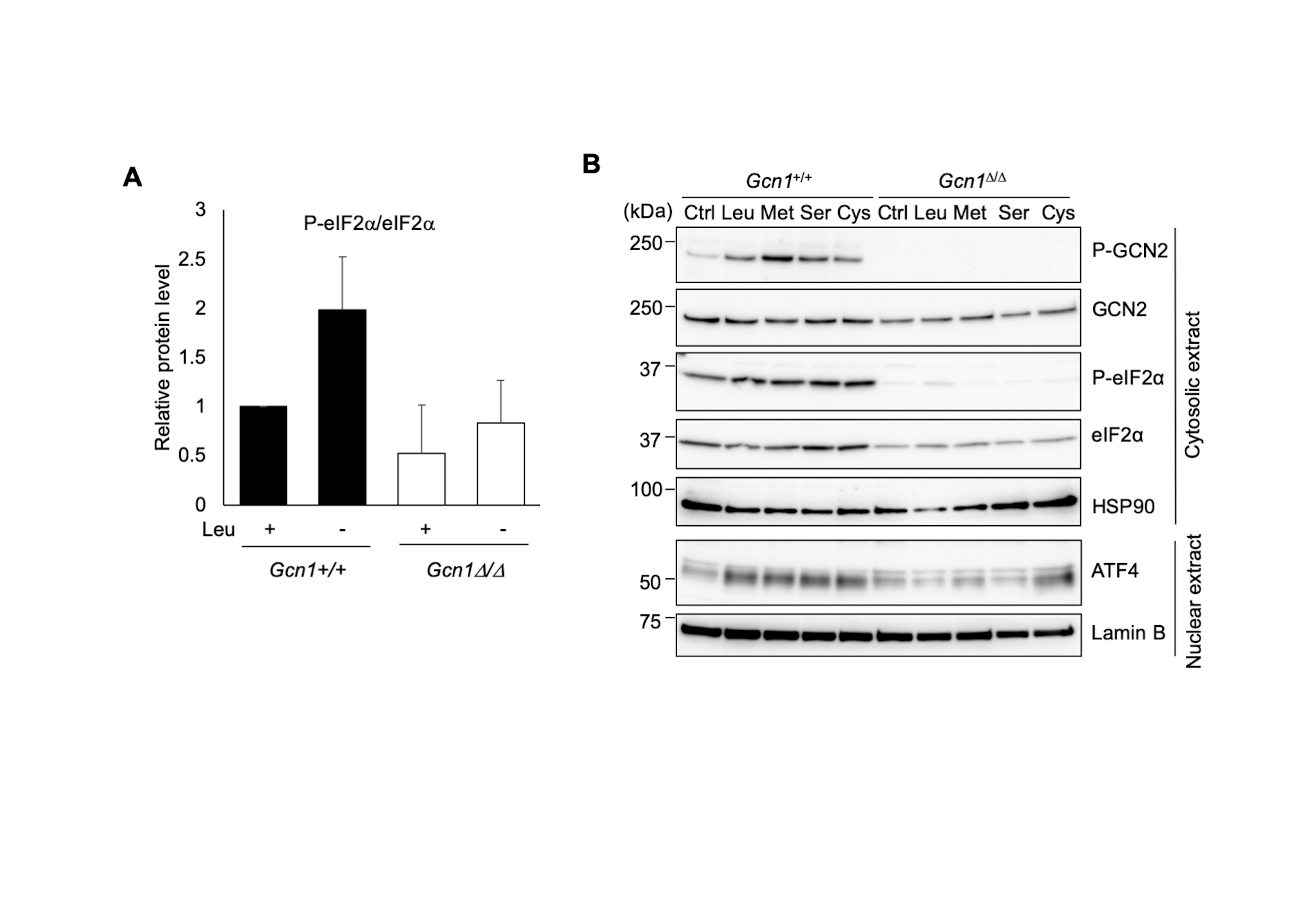

Supplement: S5 Fig — (A) The data in Fig 3B was quantified and shown. The value for the WT control was set to 1, and the results are shown as relative means±SD from multiple independent experiments (N = 3). (B) The replicate of Fig 3D was shown. The WT (Gcn1+/+) and Gcn1ΔRWDBD MEFs were exposed to leucine (Leu), methionine (Met), serine (Ser) or cystine (Cys) starvation for 4 h or cultured in the control (Ctrl) medium and cells were fractionated into cytosol, nuclear fractions and subjected to immunoblot analysis to detect the phosphorylated GCN2 (P-GCN2), GCN2, phosphorylated eIF2α (P-eIF2α), eIF2α, HSP90, ATF4 and Lamin B. (TIFF) [file pgen.1008693.s005.tiff]

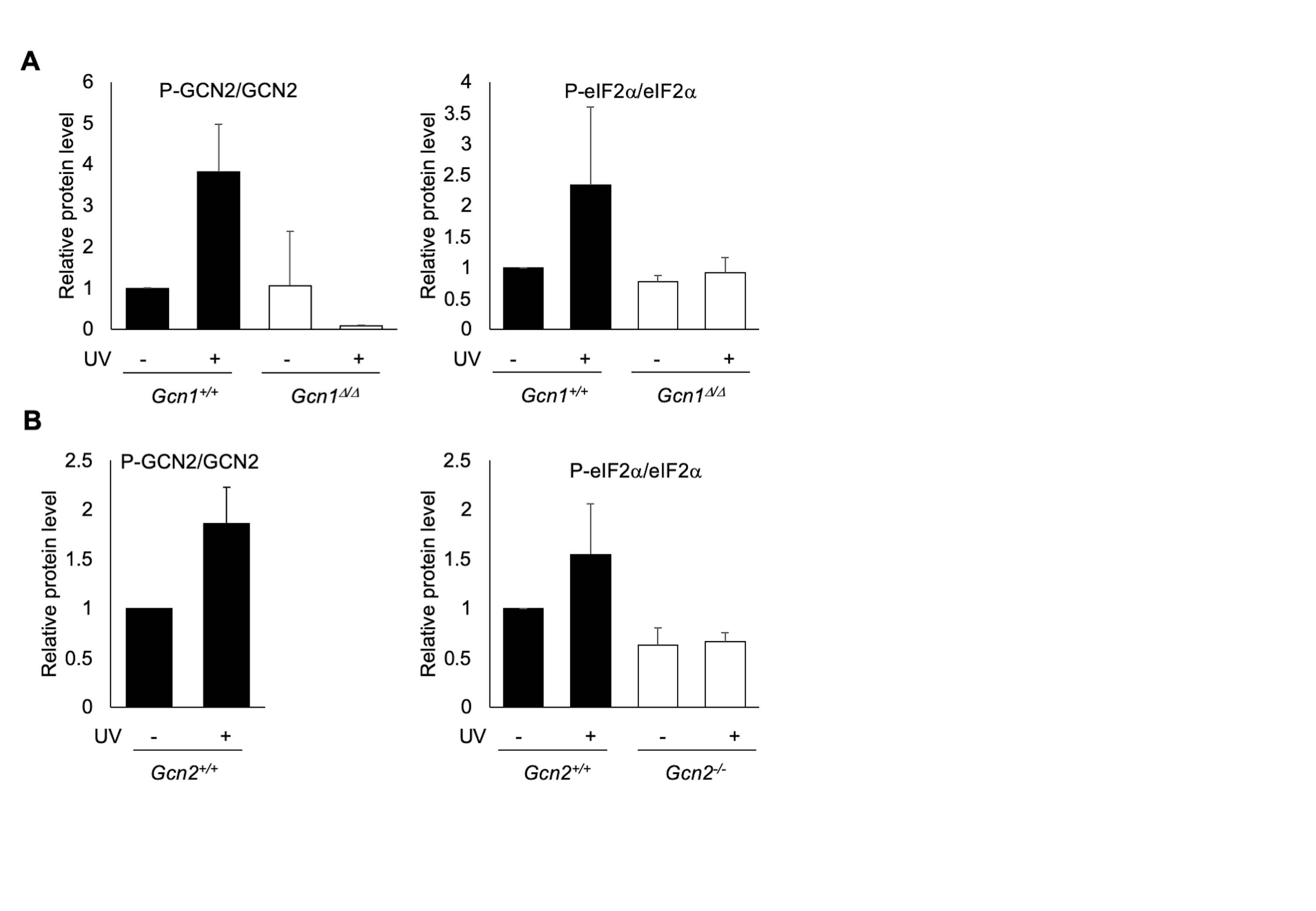

Supplement: S6 Fig — (A) The data in Fig 4A was quantified and shown. The value for the WT control cells was set to 1, and the results are shown as relative means±SD from multiple independent experiments (N = 3). (B) The data in Fig 4B was quantified and shown. The value for the WT control cells was set to 1, and the results are shown as relative means±SD from multiple independent experiments (N = 3). (TIFF) [file pgen.1008693.s006.tiff]

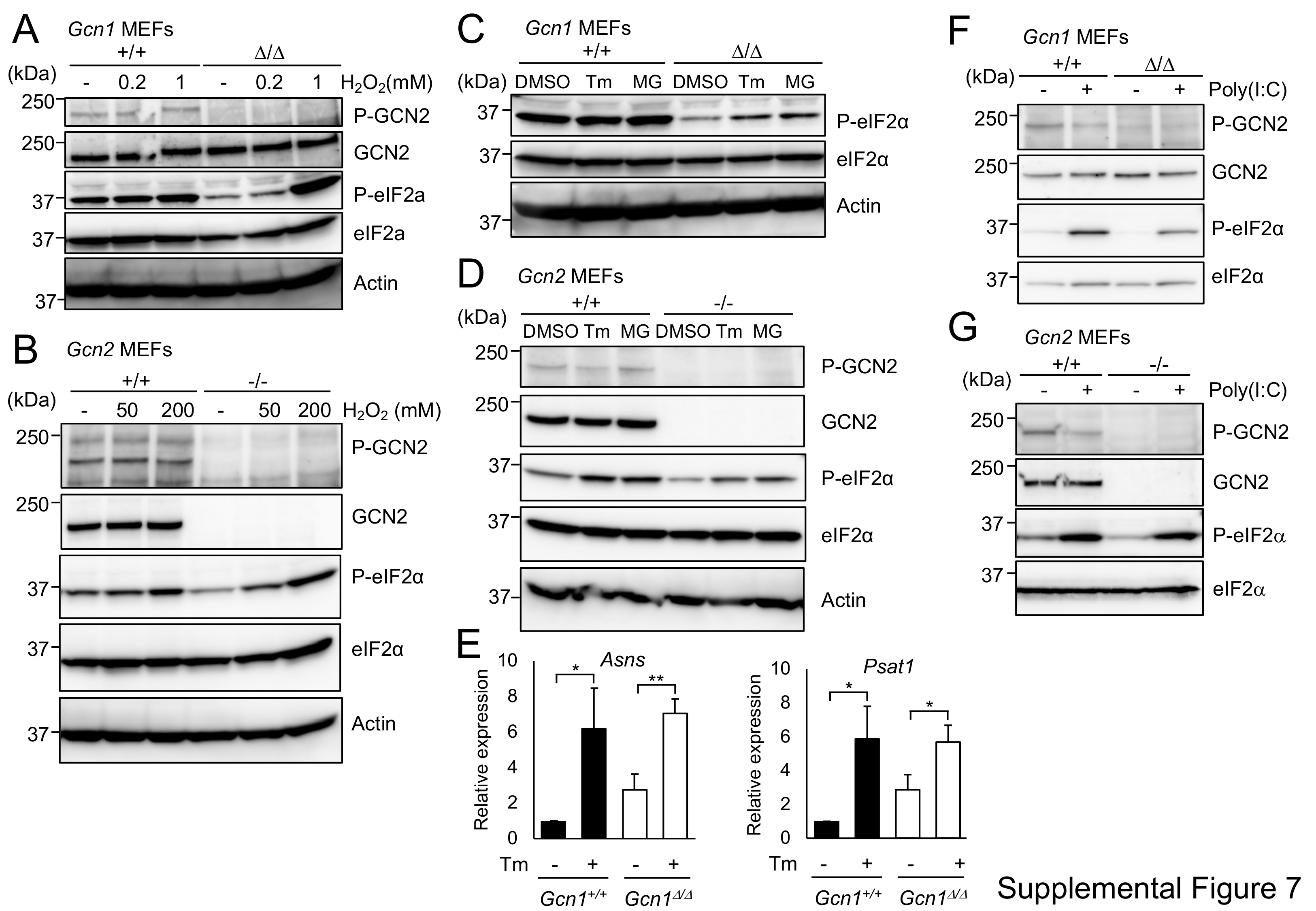

Supplement: S7 Fig — (A)(B) The WT and Gcn1ΔRWDBD (A) or Gcn2 KO (Gcn2-/-) MEFs (B) were treated by H2O2 for 1 hour and subjected to immunoblot to detect phosphorylated GCN2 (P-GCN2), GCN2, phosphorylated eIF2α (P-eIF2α), eIF2α and β-actin. (C)(D) The WT and Gcn1ΔRWDBD (C) or Gcn2 KO (Gcn2-/-) MEFs (D) were treated by 10 μM MG132 (MG) or 2 μg/mL Tm for 1 hour and subjected to immunoblot to detect phosphorylated GCN2 (P-GCN2), GCN2, phosphorylated eIF2α (P-eIF2α), eIF2α and β-actin. (E) The WT and Gcn1ΔRWDBD MEFs were treated by 2 μg/mL Tm for 16 hours, and the mRNA levels of the ATF4 target genes Asns and Psat1 were quantified by RT-PCR. The value for WT control cells was set to 1, and the results were shown as the relative folds±SD from multiple independent experiments (N = 4). * p<0.05, ** p<0.01 compared with the WT (two tailed Student’s t-test). (F)(G) The WT and Gcn1ΔRWDBD (F) or Gcn2 KO (Gcn2-/-) MEFs (G) were transfected with Poly(I:C) and incubated for 4 hour and subjected to immunoblot to detect phosphorylated GCN2 (P-GCN2), GCN2, phosphorylated eIF2α (P-eIF2α), eIF2α. (TIF) [file pgen.1008693.s007.tif]

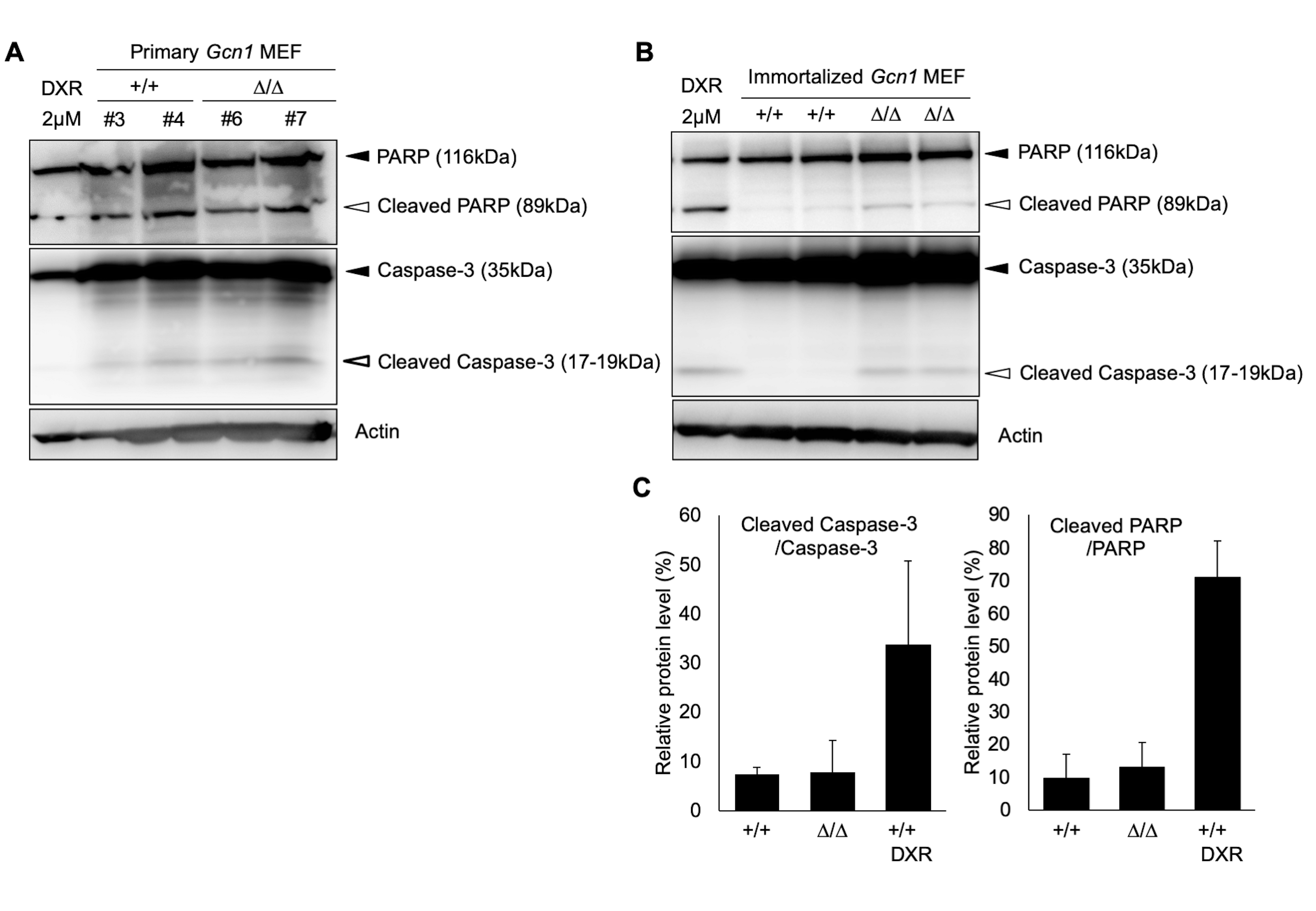

Supplement: S8 Fig — (A) Whole cell proteins extracted from WT (Gcn1+/+) and primary (A) or immortalized (B) Gcn1ΔRWDBD MEFs were subjected to immunoblot analysis to detect PARP, Caspase-3 and β-actin. Intact and cleaved forms of PARP and Caspase-3 are indicated with filled and open arrowheads, respectively. WT MEFs were treated with 2 μM doxorubicin (DXR) for 16 h and loaded as a positive control during the analysis of apoptotic cells. (C) The data in S8B Fig was quantified and shown. The results are shown as relative means±SD from multiple independent experiments (N = 4). (TIFF) [file pgen.1008693.s008.tiff]

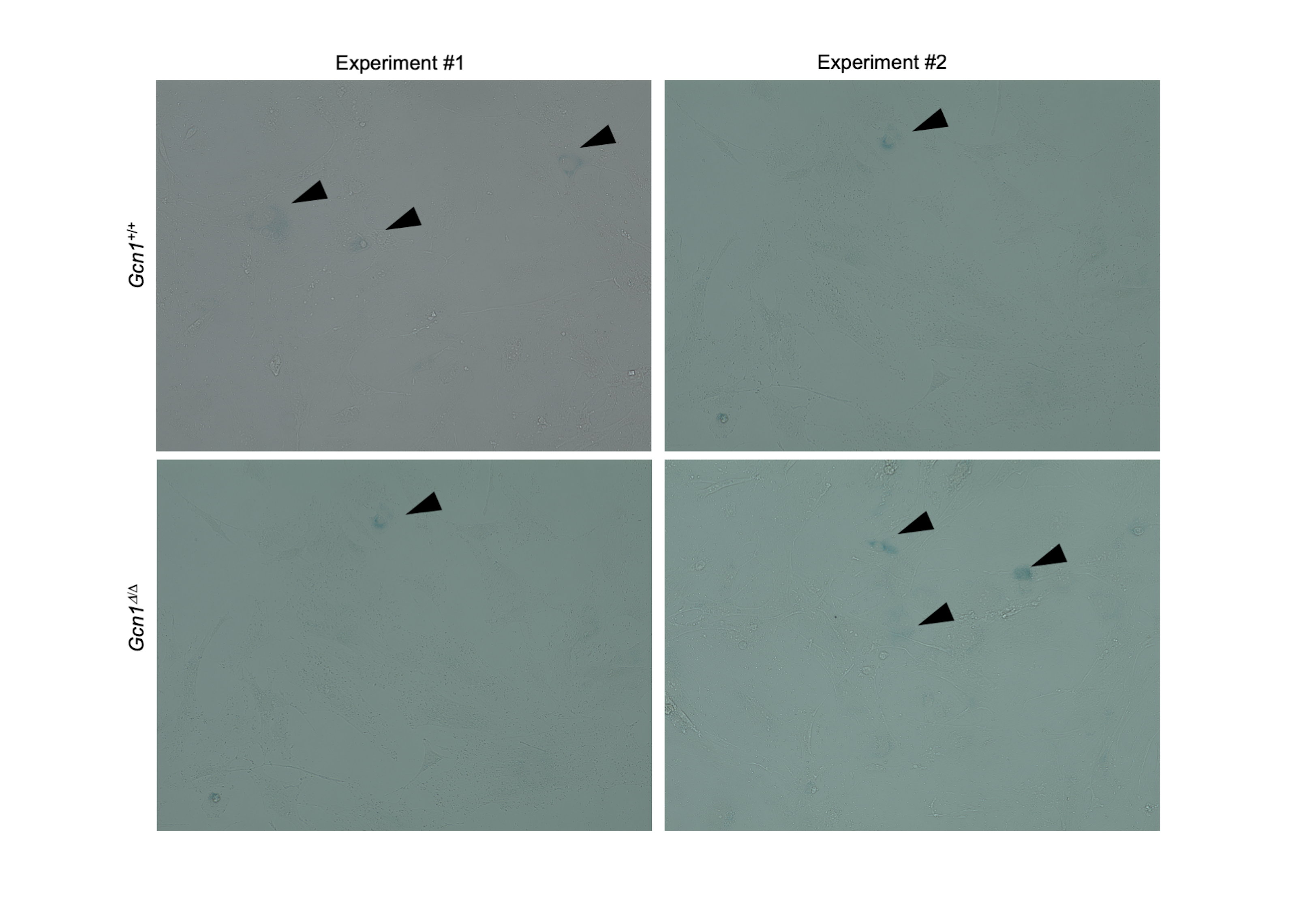

Supplement: S9 Fig — Primary WT (Gcn1+/+) and Gcn1ΔRWDBD MEFs were subjected to β-galactosidase staining and representative data was shown. Independent mouse lines were used and analyzed. The arrowheads indicate β-galactosidase positive cells. (TIFF) [file pgen.1008693.s009.tiff]

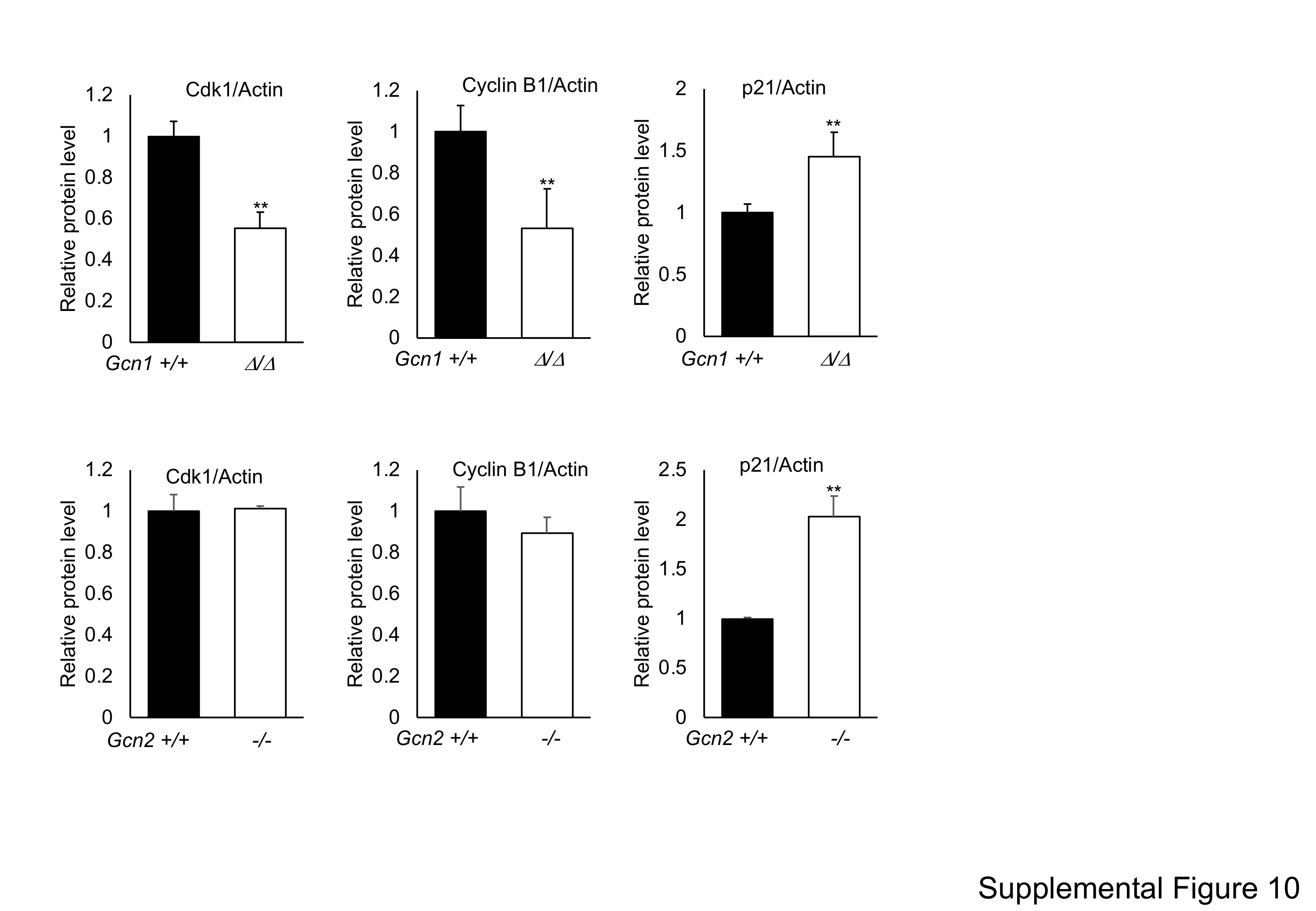

Supplement: S10 Fig — The data in Fig 6C and 6D was quantified and shown. The value for the WT was set to 1, and the results are shown as relative means±SD from multiple independent experiments (N = 3). ** p<0.01 compared with the WT (two tailed Student’s t-test). (TIF) [file pgen.1008693.s010.tif]

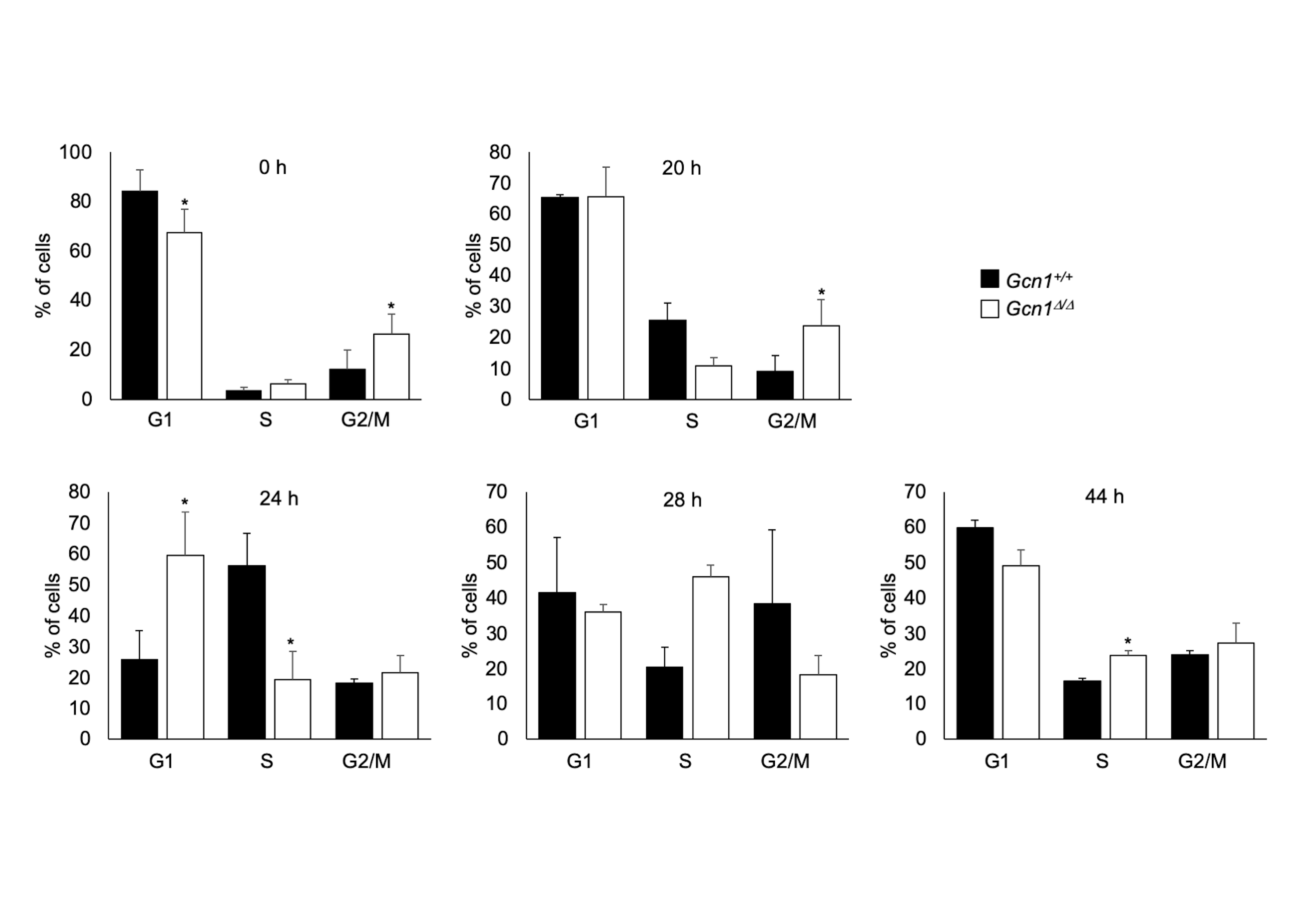

Supplement: S11 Fig — The data in Fig 6E was quantified and shown. The results are shown as relative means±SD from multiple independent experiments (28 h: N = 2, 0 h, 20 h, 24 h and 44 h: N = 3). * p<0.05 compared with the WT (two tailed Student’s t-test) (statistical analysis was not performed at 28 h). (TIFF) [file pgen.1008693.s011.tiff]

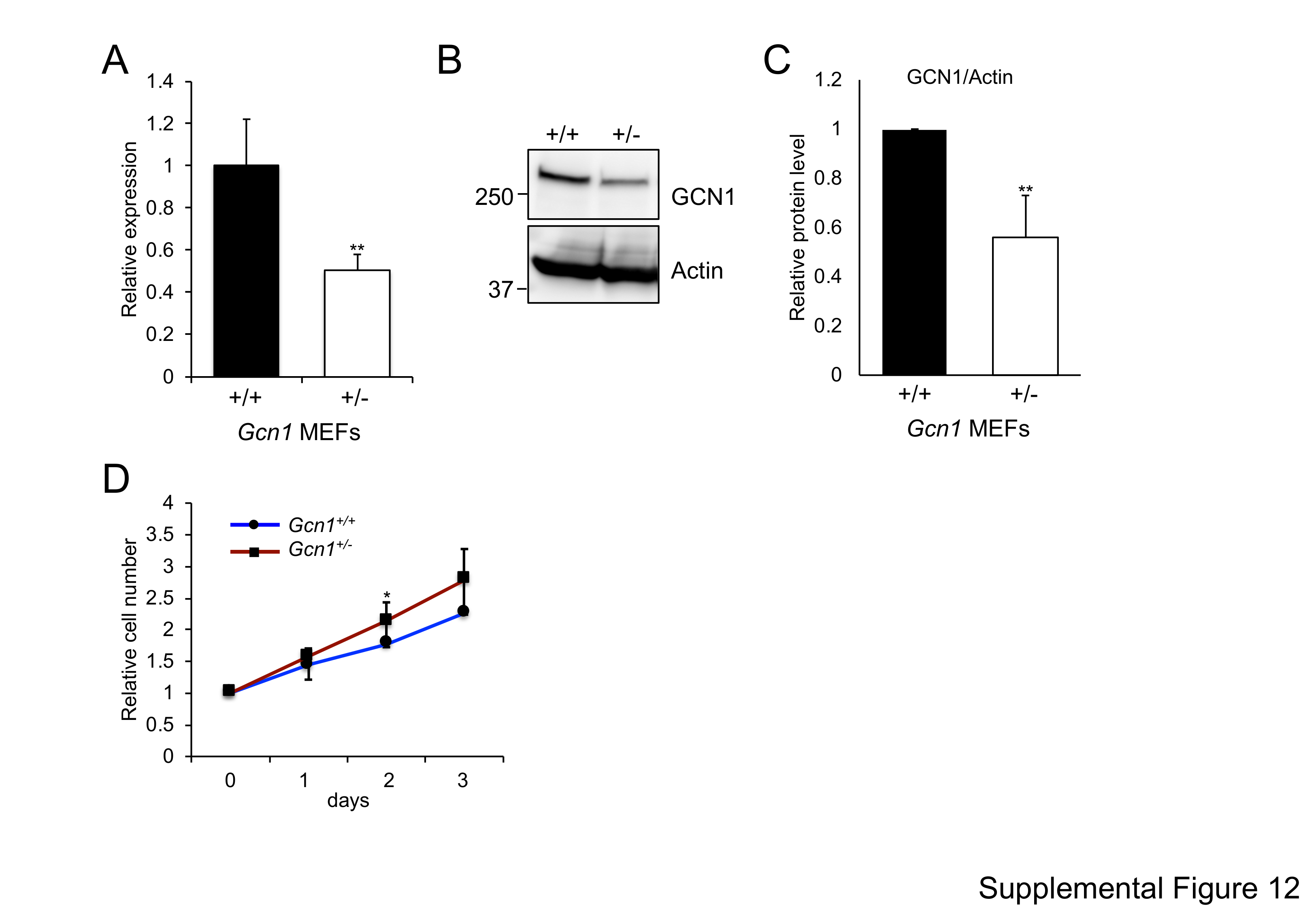

Supplement: S12 Fig — (A) Gcn1 expression levels in the WT (Gcn1+/+) and heterozygous Gcn1 KO MEFs were quantified by RT-PCR. Primers (spanning exon 2 to 3) were designed to detect WT mRNA but not mRNA from Gcn1 KO allele. The value for wild-type cells was set to 1, and the results are shown as the relative folds±SD from multiple independent experiments (N = 3). ** p<0.01 compared with the WT (two tailed Student’s t-test). (B)(C) Whole cell protein extracted from the WT (Gcn1+/+) and heterozygous Gcn1 KO MEFs was subjected to immunoblot analysis to detect GCN1 and β-actin. The data in S12B Fig was quantified and shown in (C). The value for wild-type cells was set to 1, and the results are shown as the relative folds±SD from multiple independent experiments (N = 3). ** p<0.01 compared with the WT (two tailed Student’s t-test). (D) After cells were cultured in IMDM for the indicated periods, the relative cell numbers of primary heterozygous Gcn1 KO MEFs were counted and are shown with the corresponding WT (Gcn1+/+) cells. The initial cell number was set to 1 and the results are shown as the relative folds±SD from multiple independent experiments (N = 3). * p<0.05 compared with the WT (two tailed Student’s t-test). (TIF) [file pgen.1008693.s012.tif]

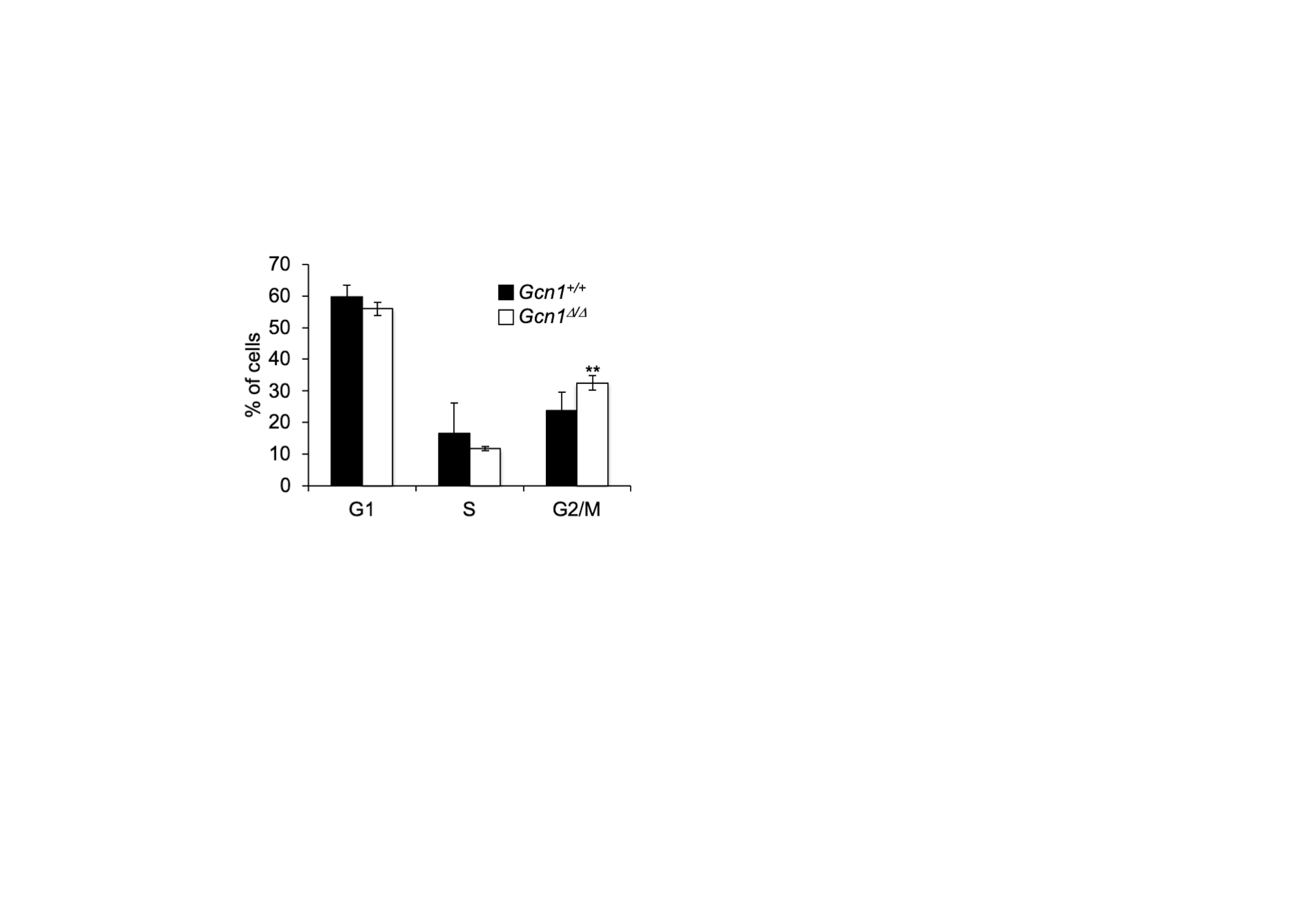

Supplement: S13 Fig — The WT (Gcn1+/+) and Gcn1ΔRWDBD immortalized MEFs were stained by propidium iodide (PI) to assess the DNA contents in different phases of the cell cycle and the percentage of cells in each cell cycle was calculated. The results are presented as fold differences compared to those of the WT (Gcn1+/+) MEFs from multiple independent experiments (N = 6). ** p<0.01 compared with the WT (two tailed Student’s t-test). (TIFF) [file pgen.1008693.s013.tiff]
