# Supplementary material for: Ribosome binding protein GCN1 regulates the cell cycle and cell proliferation and is essential for the embryonic development of mice
Source: PLoS Genet. 2020 Apr 23;16(4):e1008693. doi: 10.1371/journal.pgen.1008693 (PMC7179835; doi:10.1371/journal.pgen.1008693)
Supplement: Attachment — Submitted filename: Yamazaki_et_al_Response_to_Reviewers_F.pdf [file pgen.1008693.s014.pdf]

We thank the reviewers for the constructive comments. We revised the manuscript and figures according to the reviewers' comments. Also, according to the editor's suggestion we removed "data not shown" in the manuscript as shown below (No.1 to 6).

1. P.6 These founder mice were mated with wild-type (WT) mice, and 4 independent lines were established. *Gcn1*$^{+/\Delta RWDBD}$ mice were viable and fertile (data not shown).

→We deleted "data not shown".

2. P.6 Although growth retardation was observed, the *Gcn1*$^{\Delta RWDBD}$ embryos were apparently normal, although some of these embryos showed abnormalities in the head or had an anencephaly-like phenotype at E14.5 (data not shown) and E18.5 (Fig 1I).

→We added the representative pictures of *Gcn1*$^{\Delta RWDBD}$ embryo that showed abnormality at E14.5 as Fig S2A.

3. P.7 At E20.5, we observed 5 *Gcn1*$^{\Delta RWDBD}$ embryos with normal appearance and 4 *Gcn1*$^{\Delta RWDBD}$ embryos with mandibular hypoplasia and exophthalmos/hypoplasia of the eyelid (Fig 2C and data not shown).

→We inserted the representative pictures of *Gcn1*$^{\Delta RWDBD}$ embryo with mandibular hypoplasia and exophthalmos/hypoplasia of the eyelid at E20.5 in Fig S2B and C.

4. P.7 The *Gcn1*$^{\Delta RWDBD}$ embryos with abnormal appearance died within 20 min after delivery by cesarean section, whereas 2 of 5 *Gcn1*$^{\Delta RWDBD}$ embryos with normal appearance respired, turned red, and survived for at least 4 h (Fig 2C and data not shown).

→We deleted "data not shown".

5. P.12 *Gcn1* heterozygous KO MEFs expressed about half amount of mRNA and protein compared with WT MEFs (Fig S12A-C). And heterozygous *Gcn1* KO mice showed neither perinatal lethality nor embryonic growth defects (data not shown and Fig S12D), suggesting that at least one-half the amount of GCN1 is sufficient for normal development.

→We deleted "data not shown".

6. P. 13 We observed growth defects and an increase in the G2/M population in both immortalized and primary *Gcn1*$^{\Delta RWDBD}$ MEFs (Fig 5A, B, E, F and data not shown).

→We added the figure of cell cycle analysis using WT and *Gcn1*$^{\Delta RWDBD}$ immortalized MEFs as Fig S13.

Reviewer #1: In this article authors have produced, by using CRISPR/Cas9 technique, the first mice devoid of the function of GCN1L1 protein, the mammalian homolog of budding yeast protein Gcn1. Gcn1 has been shown to be non-essential for yeast cells, but necessary for the activation of the stress responsive eIF2α kinase Gcn2, also present in mammalian cells. Authors have obtained two types of mutant mice, one with no expression of GCN1L1, and the other expressing a truncated form of the protein which is supposed to be unable to interact with its main target GCN2. Interestingly, and unlike the GCN2 KO, both mice present a severe phenotype of growth retardation at the embryonic stage mainly affecting pulmonary development and maturation, suggesting an essential and GCN2-independent role for GCN1L1 in mammals.
Consequently, cells derived from these mice show diminished growth and GCN2-dependent stress response, but also additional cell cycle abnormalities absent in GCN2 KO cells.

The manuscript is well organized and clearly written, and, in general, data and analyses support the conclusions. Nevertheless, below there are a series of concerns:

Response: We thank the reviewer for the constructive and straightforward comments.

Introduction
Ref. #5, studying PKR sensitivity to viral inhibitors, does not illustrate the authors′ statement ″GCN2 might be the most ancient elF2α kinase in eukaryotes found in yeasts, plants and mammals, and budding yeast Saccharomyces cerevisiae (S. cerevisiae) possess Gcn2 as the sole elF2α kinase (5)″

Response: Thank you for the comment. We replace the Ref. #5 with the paper by Krishna KH et al.

Ref. #9 is a paper done by using the fission yeast Schizosaccharomyces pombe (S.

pombe) instead of S. cerevisiae, as authors seem to claim: "In S. cerevisiae, uncharged tRNA binding to Gcn2 is essential for the response to AAS as well as to hydrogen peroxide and UV radiation, and these responses require Gcn1 (9)" I suggest to substitute reference #16 (Murguía and Serrano, 2012), because it is a review, for the original one demonstrating an additional substrate for GCN2 kinase: Kwon NH, Kang T, Lee JY, Kim HH, Kim HR, Hong J, Oh YS, Han JM, Ku MJ, Lee SY, Kim S. Dual role of methionyl-tRNA synthetase in the regulation of translation and tumor suppressor activity of aminoacyl-tRNA synthetase-interacting multifunctional protein-3. Proc Natl Acad Sci U S A. 2011:108(49):19635-40. doi: 10.1073/pnas.1103922108.

Response: Thank you for the comment. According to the reviewer's comment, we changed the sentences referring to Ref. #9 and also added the explanation.

Results

Page 6 (bottom): "...and showed a statistically significant decrease in body weight after E17.5 (Fig 2C)", I guess figure referred is 2B instead of 2C.

Response: We appreciate for pointing out the mistake. We have corrected the figure number.

Page 7 (end of first paragraph): "...and the gene expression levels of alveolar markers were comparable to those of the WT embryos between E18.5 and E19.5 (Fig 2E and F)". Even though this sentence is not relevant for the global conclusion of the work, it does not reflect what it can be observed in the referred figure. According to the graphs in Fig. 2F, there is a significant difference in Aqp5 and Sp-c mRNA expression levels at E18.5 and E19.5.

Response: Thank you for the comment. We wanted to describe that the gene expression levels of alveolar markers of E20.5 Gcn1l1$^{\Delta RWDBD}$ were comparable to those of the WT embryos of E18.5 (Fig 2E and F). We improved the description in the revised manuscript.

Pages 7 (bottom) and 8 (top): most of the referred results regarding subcellular localization appear in the supplementary figure 2, but they have enough interest to be part of a main figure. Nevertheless, I would suggest to use MEF instead of HeLa cells and compare between WT and Gcn1l1ΔRWDBD cells, given that subcellular localization of the protein could be relevant for its function.

Response: We tried to examine the GCN1L1 subcellular localization in MEFs but we could not find suitable immunostaining conditions to detect GCN1L1 in MEFs. Instead

of immunostaining, we performed western blot analysis of MEFs samples fractionated to nucleus and cytoplasm (Fig S3E). In both WT and Gcn1l1 ΔRWDBD MEFs, GCN1L1 mainly localized to the cytoplasm similarly to HeLa cells.

Page 8 (middle paragraph): the phospho-specific western blot and the RT-PCR experiments strongly support the authors′ statement, but they would also consider to directly measure the subsequent increase in ATF4 translation upon eIF2α phosphorylation, by western blot.

Response: We measured the nuclear accumulation of ATF4 and found that ATF4 accumulation was impaired in Gcn1l1 ΔRWDBD MEFs upon amino acid starvation (Fig 3D and Fig S5B).

Page 8 (bottom paragraph): results shown in figure 4A do not support authors′ statement, at least after 4 h of UV treatment. While GCN2 phosphorylation does not increase in WT cells and it is absent in mutant cells, it seems like eIF2α phosphorylation increases in both cell types. A reference to repeats or statistics is not present in the figure legend.

Response: Thank you for the comment. We interpreted the results that the increase of eIF2α phosphorylation at 30 minutes after UV radiation is dependent on both GCN2 and GCN1, but those at 4 hours are only partially dependent on GCN1-GCN2 pathway. At 4 hour time point, eIF2α kinase other than GCN2 is activated or eIF2α phosphatase is inhibited most probably due to the indirect effect of UV radiation. To avoid confusion, we focused on the 30min and analyzed the results repeatedly. P-GCN2/GCN2 and P-eIF2a/eIF2a were quantitated and shown in Fig S6.

Page 9 (top paragraph): results regarding differences in cell growth and BrU incorporation seem to be clear, but the argued differences in cell size and in cell cycle seem to be not very prominent, even though statistically significant in some cases. Another question that could be apparently contradictory is the fact that with lower BrU incorporation mutant cells showed increased proportion in S and G2/M cell cycle phases.

Response: We appreciate for the comment. We agree with the reviewer that differences in cell size and in cell cycle is not very prominent, although they are statistically significant. Regarding to the latter comment, we surmise that if the cells are arrested in

G2/M with resultant increase of S phase population, the incorporation of BrdU could be diminished because of the decreased flux into the S phase.

Page 10, lines 7, 8, 9: in line 7 it should be "Fig. 6C and D" instead of Fig. 6C and 5D); in line 8 it should be "Fig. 6C" instead of "Fig. 6A"; in line 9 it should be "Fig. 6A" instead of "Fig. 6C"

Response: We are sorry for the mistake. We have corrected the figure number.

Discussion

Page 11, line 6 it should be "Fig. 1 and 2" instead of "Fig. 1 and B"

Response: We are sorry for the mistake. We have corrected the figure number.

Page 11, end of the first paragraph: authors state that heterozygous GCN1L1 KO mice express half amount of protein compared with wild type, but they do not experimentally demonstrate this point.

Response: Thank you for the comment and we think it is an important point. We have added the data that heterozygous Gcn1 KO MEFs (i.e. Gcn1 $^{+/\Delta exon2}$ MEFs) express about half amount of mRNA and protein compared with WT in Fig S12A-C.

Page 13, first paragraph: authors argue that similarities between OLA1 and GCN1L1 KO mice and cells could be due to the reduction in ternary complex levels induced by both proteins in normal cells. This observation suggests that GCN1L1 function, although GCN2-independent, could still be related with eIF2α phosphorylation (reduced in mutant cells), raising the following question: is it possible that GCN1L1 could also regulate the activity of any of the other three eIF2α kinases (PKR, PERK, HRI)? Then, maybe authors should check this possibility by analysing the effect of the lack of GCN1L1 in the activation of those eIF2α kinases.

Response: Thank you for the comments and we agree with the reviewer's comment. As we do not possess any evidence that support the contention of the discussion, we corrected the original sentence to "Although both OLA1 and GCN1 can mediate stress-induced decreases in ternary complex formation, the reasons for similarity should be clarified in the future". This having said, we sometimes observed that eIF2α phosphorylation is very low at normal culture conditions in Gcn1l1$^{\Delta RWDBD}$ MEFs compared to wild-type MEFs. Therefore, we examined the effect of GCN1L1 deletion on the eIF2α phosphorylation activated by other three eIF2α kinases and presented them as supplemental Figure S6.

Reviewer #2: Yeast GCN1 is a multi-domain protein that associates with the RWD portion of GCN2 protein kinase, contributing to its activation and phosphorylation of eIF2 in response to amino acid depletion. Although GCN2 in mammalian cells has been well documented to induce the Integrated stress response in response to nutrient stresses, the role of the mammalian GCN1 ortholog (GCN1L1) has only sparsely studied. This manuscript addresses the function of GCN1L1 using mouse knockout mouse and in culture using MEF cells. The key findings of the manuscript are that loss of GCN1L1 function leads to a mouse growth defect that appears to involve delayed lung function. This growth defect is suggested to be independent of GCN2 as no growth defect has been reported for GCN2-deleted mice. Furthermore, this manuscript shows that mice expressing only GCN1l1 devoid of its GCN1-binding domain (RWD-BD) do not show the growth defect. Using MEF cells derived from this GCN1-modified mice, the manuscript shows that both GCN1 mutations thwart GCN2 activation in response to different stresses. There is also a cell cycle defect with loss of GCN1L1 that is appears to be independent of GCN2. Overall, these studies largely support the model presented in figure 7.

These results indicate that mammalian GCN1L1 not only functions in the activation of GCN2 (similar to yeast) but also has additional functions developmental and proliferative functions that are likely independent of GCN2. These findings are significant to the field and of broad interest. The manuscript is clearly written (although it should include more paragraph breaks) and experiments and appropriately designed and interpreted. There is a lot of enthusiasm for this manuscript, with only minor concerns.

Concerns:

1. Statistical analyses appear to be appropriate although there could be additional information (t-test one or two tailed?; Annova one or two way? Number of biological replicates?).

Response: Thank you for the comment. We have added detailed information.

2. MW marker designation should be included in immunoblots. Quantitation with associated statistics should be included for key immunoblot measurements.

Response: MW marker designation was included in immunoblots. Also, we have measured the band intensity of the main important blots (Fig 3B, 4A-B, 6C-D) and presented them as supplemental Figures (Fig. S5A, Fig. S6A-B, Fig. S10, respectively).

3. The last sentence in the results subsection titled "GCN1L1 was also necessary for the GCN2-mediated response to UV stress" describes Gcn1l1 KO MEFs for Fig 4A and 4B (should only be 4A . The figure 4 legend should also be clearer regarding panels A and B.

Response: We interpreted the results of the previous UV experiment that the increase of eIF2α phosphorylation at 30 minutes after UV radiation is dependent on both GCN2 and GCN1, but those at 4 hours are only partially dependent on GCN1-GCN2 pathway. At 4 hour time point, eIF2α kinase other than GCN2 is activated or eIF2α phosphatase is inhibited. To avoid confusion, we focused on the 30min and analyzed repeatedly. P-GCN2/GCN2 and P-eIF2a/eIF2a were quantitated and shown in Fig S6. Accordingly, we improve the last sentence in the results subsection titled "GCN1 was also necessary for the GCN2-mediated response to UV stress"

4. The proposed mechanism for the observed p21 increase in Gcn1l1ΔRWDBD and Gcn2 KO MEF cells presented in the discussion section appears to have an error. The second to last paragraph of the discussion section begins by proposing that p21 protein levels are increased by a decrease in ternary complex. However, ternary complex levels would be increased rather than decreased upon loss of eIF2 phosphorylation. This proposed mechanism should more clearly explain the observed increase in p21 levels that result from Gcn1l1ΔRWDBD or Gcn2 KO.

Response: We are sorry for the mistake. We corrected the sentence "p21 protein levels are increased by a decrease in ternary complex" to "p21 protein levels are increased by a decrease in ternary complex". Furthermore, p21 mRNA levels can be increased both by GCN2 loss and the DRG2 loss. We clarified our hypothesis in the "Discussion section" and also we corrected the Figure 7.

5. Deletion of the RWD binding domain of GCN1L1 in MEF cells reduced the total expression of GCN1L1 by greater that fifty percent (shown in Fig 3A) – therefore it is not entirely clear whether the resulting slowed growth, decreased Cdk1/cyclin B1, and increased p21 levels are a result of this decrease in expression or a result of the deletion of the RWD domain.

Response: I appreciate for the reviewer's comment. We agreed with the reviewer and fully discussed about this in the first paragraph of the Discussion section.

6. The decrease in cellular proliferation observed in Gcn1l1ΔRWDBD is not clearly attributed to either decreased Cdk1/cyclinB1 or increased p21. The manuscript cites both the increase in G2/M phase population (Fig. 5F) and the delay in entering S phase (Fig. 6E) of Gcn1l1ΔRWDBD MEFs as evidence for the decreased proliferation observed in Fig. 5A. Gcn1l1ΔRWDBD and Gcn2 KO both exhibit elevated p21 mRNA and protein levels, and while the Gcn1l1ΔRWDBD MEFs experienced a decrease in proliferation, the Gcn2 KO MEF cells did not. The delay in entering S phase observed in Fig 6E could be a result of increased p21 levels in Gcn1l1ΔRWDBD cells as compared to wild-type. Therefore, it may be necessary to address whether Gcn2 KO MEFs do not exhibit this delay in S phase entry, if in fact the delay in S phase contributes to reduced proliferation observed in Gcn1l1ΔRWDBD cells.

Response: Thank you for the comment and we agree that "the decrease in cellular proliferation observed in Gcn1l1ΔRWDBD is not clearly attributed to either decreased Cdk1/cyclinB1 or increased p21". We would like to clarify this point in the future analysis. Furthermore, one caveat in this study is that we used GCN2 KO MEFs transformed by large T antigen. We tried to obtain primary GCN2 KO MEFs to formally compare the proliferation phenotype of GCN2 KO MEFs and GCN1 ΔRWDBD cells, but we could not get primary GCN2 KO MEFs. We described this caveat in the Discussion section. We would like to clarify the exact mechanisms of the decreased proliferation in Gcn1l1ΔRWDBD cells in future analysis.

7. Perhaps it should be noted that humans with GCN2 defects have lung injuries.

Response: We noted that the GCN2 defect causes pulmonary hypertension in human in the Introduction section referring to the paper by Eyries M. et al.

Reviewer #3: There was some previous evidence that mammalian Gcn1 (mGcn1) is required for activation of mGcn2 based on overexpressing the IMPACT protein in mammalian cells, which competes for the RWD domain in mGcn2 for binding to mGcn1 and blunts the activation of mGcn2 and eIF2 phosphorylation in response to leucine starvation or UV treatment. Importantly, however, this paper provides direct evidence that Gcn1, and most likely its RWD binding domain (RWDBD), is required for Gcn2 activation in response to these stresses. They created two mGcn1 mutant mouse lines, one designed to knock out the entire protein and the other to eliminate only the RWDBD. Interestingly, these two mutant mice showed growth retardation and lethality, which was not observed for the Gcn2 KO mice, suggesting that mGcn1 has additional roles other than activation of Gcn2, and is essential for embryonic development. They also found reduced cell proliferation and G2/M arrest accompanying

a decrease in Cdk1 and cyclinB1 in the mGcn1 ΔRWDBD MEFs, again not seen in mGcn2 KO MEFs, further suggesting a GCN2-independent role for mGcn1 in cell cycle regulation. Although the molecular mechanism involved in promoting expression of Cdk1 and cyclinB1 by mGcn1 remain to be determined, including which other RWD-containing protein is the relevant interaction partner for mGcn1 in the mechanism, the results are interesting and important nonetheless in uncovering this alternative function for mGcn1 in addition to providing strong, direct evidence that mGcn2 binding to the RWDBD of mGcn1 is required for Gcn2 activation by amino acid starvation and UV stress.

Major comments:

-p. 8. The statement: "Both the Asns and Psat1 genes were induced by depletion of methionine, serine or cystine in the WT MEFs, whereas the induction was largely suppressed in both Gcn1l1ΔRWDBD and Gcn2 KO MEFs (Fig 3D and E)." does not appear to be justified by the lack of a statistically significant reduction of Asns and Psat1 expression in the ΔRWDB mutant, and further evidence is required to claim that mGcn1 mediates the response to starvation for amino acids other than Leucine. One approach, which would enhance the story significantly would be to show that increased expression of Atf4, or an Atf4 reporter in response to different amino acid limitations in impaired in the ΔRWDBD MEFs.

Response: Thank you for the comment. Accordingly, we measured the nuclear accumulation of ATF4 and found that ATF4 accumulation was impaired in Gcn1l1 ΔRWDBD MEFs upon amino acid starvation (Fig 3D and Fig S5B).

-Based on the previous findings that In DRG2-knockdown HeLa cells, CDK1 and Cyclin B1 are decreased, while p21 is increased, the authors suggest in their model that Gcn1 functions in concert either DRG2 in stimulating Cdk1-cyclinB1 expression. If so, then knocking down DRG2 in the ΔRWDBD MEFs should not lead to any additional reduction in CDK1 and Cyclin B1 levels, but should do so in Gcn1+/+ MEFs. Performing this additional experiment has the potential of increasing the impact of this paper significantly by placing Gcn1 and Drg2 in the same pathway in regulating cell cycle, and as such, is strongly recommended.

Response: Thank you for the comments and we agree with the reviewer's comment. Accordingly, we knocked down DRG2 in both wild-type and ΔRWDBD MEFs. However, knockdown efficiency is too low to analyze the effect of DRG2 on Cdk1-cyclinB1 expression. Therefore, we would like to clarify the issue in the future analysis.

Other comments:

-p.6, line 7 from bottom: In correct citation; should be Fig. 2B instead.

Response: We have corrected the figure number.

-p.7, lines 2-6: the legend to Fig. 2B lacks information that some of the data apparently came from experiments in which the gestational period was increased but other data did not; and this needs to be clarified.

Response: Thank you for the comment. We have added the detailed explanation.

-Fig. 2D-F: information is regarding the exact number of different animals of each genotype were examined and showed the indicated lung abnormalities or lung marker expression differences; and the statistical test applied in panel F.

Response: We have corrected the manuscript.

-Fig. 3A lacks a control for equal protein loading.

Response: Thank you for the comment. We have added the loading control in new Fig. 3A.

-Fig. 3B lacks information about how many replicate experiments were conducted that gave a similar result.

Response: We conducted 3 replicate experiments and the quantified data was inserted in Fig S5A.

-Fig. 3C-E lacks information about whether the replicates are biological replicates from independent RNA isolations or just technical replicates of the PCR amplifications of the same RNA samples.

Response: The results were from multiple independent experiments and we have corrected the manuscript and added the number of replicated experiments.

-p.8 and Fig. 4B: Either replicate blots should be presented as supplementary material or (even better) quantification of blots from biological replicates should be provided for the P-eIF2a:eIF2a ratios to justify the conclusions that the ΔRWDBD mutant and the Gcn2 KO both reduce the proportion of eIF2a that is phosphorylated in response to UV.

Response: Thank you for the comments. We surmise that the increase of eIF2α phosphorylation at 30 minutes after UV radiation is dependent on both GCN2 and GCN1, but those at 4 hours are only partially dependent on GCN1-GCN2 pathway. At 4 hour time point, eIF2α kinase other than GCN2 is activated or eIF2α phosphatase is inhibited. To avoid confusion, we focused on the 30min and analyzed repeatedly. P-GCN2/GCN2 and P-eIF2a/eIF2a were quantitated and shown in Fig S6.

-p. 8, 3rd line from bottom: cites Gcn1 KO incorrectly; should be Gcn2 KO according to Fig. 4B.

Response: We have corrected the manuscript.

-Fig. 5C,D,F,and I: the number of replicates and the statistical tests employed should be indicated in the legend. Also, in panels C-D it is unclear if primary or immortalized MEFs were analyzed and should be stipulated in the legend.

Response: Thank you for the comment. We have corrected the manuscript.

-In Fig. S4B, it's unclear if the amount of cleaved PARP and caspase-3 is increased relative to the uncleaved species, which also were higher in the mutant cells, indicating a need for quantifying the cleaved to full-length ratios from multiple replicates to justify the claim on p. 9. Also what would be the reason for seeing the increased cleavage only in the immortalized cells?

Response: Thank you for the comments. We measured the cleaved PARP/PARP and cleaved Caspase-3/Caspase-3 ratio and found that there was no difference between WT and Gcn1l1ΔRWDBD MEFs. We erased the phrase "However, these levels were slightly increased in the immortalized Gcn1l1$^{\Delta RWDBD}$ MEFs compared to the WT MEFs (Fig S8C)." We are sorry for the inadequate analysis.

-the statement on p. 9: "Cell enlargement is often associated with cellular senescence (27); however, β-galactosidase staining showed similar intensities in both WT and Gcn1l1ΔRWDBD MEFs (data now shown)" needs to be backed up by showing the data and also explained in regard to whether or not the mutant cells show signs of senescence.

Response: The data was added as Fig S9.

-p. 10, lines 2-5: It's consistent with the data but not demonstrated by the results in Fig. 6A-B that Gcn1 is involved in the regulation of p21 expression by Gcn2.

Response: Thank you for the comment and we are sorry for the overstatement. We deleted the portion “indicating that GCN1 regulates cell cycle-related genes through both GCN2-dependent and GCN2-independent pathways” and discussed it in the second last paragraph in the Discussion section in detail.

-p. 10 and Figs. 6C, D, F: Either replicate blots should be presented as supplementary material or quantification of blots from biological replicates should be provided to justify the conclusions on p. 10 regarding the altered expression of Cdk1, cyclin B1, p21, and eIF2a proteins.

Response: We have measured the band intensity and quantified data were added as Fig S10.

-p. 10: the statement: "Replenishment with fresh medium resulted in more WT MEFs in the S phase at 24 h, but at 28 h, fewer Gcn1l1ΔRWDBD MEFs and Gcn1l1ΔRWDBD MEFs were in the S phase compared to WT MEFs (Fig 6E)." has an obvious typo. In addition, it appears that there may be as many or more, not less, S-phase cells at 28h in the mutant vs. WT, and there are fewer mutant vs. WT cells in the G2/M phase at this time point. Also the data in Fig. 6E should be quantified from replicates as was done for Fig. 5F & I.

Response: The data in Fig. 6E was quantified from replicates and shown in Fig S11.

-It's unclear why the data in Fig. 5E-F and 6E seem to give conflicting results about whether there is a delay at G2/M (Fig. 5) or in S (Fig. 6).

Response: Thank you for the comment and I understand the reviewer’s concern. One explanation for the discrepancy is that the delay in the S phase might be masked by the delay in G2/M in the normal culture condition. Other possibility is that the serum starvation experiment requires the response to acute signal from the growth factors, but the normal culture condition only requires the response to the chronic growth stimuli. We would like to clarify the issue in the future analysis.

-Fig. 7A, the fact that the Gcn1-/- embryos die should be indicated

Response: We have corrected the figure.

-p. 13: The sentence: "As both OLA1 and GCN1L1 can mediate stress-induced decreases in ternary complex formation, the phenotype observed for Gcn1l1ΔRWDBD may reflect the overlapping function of OLA1 and GCN1L1. As Gcn2 KO does not show a similar phenotype, such stresses, if any, are not likely conferred by AAS." Doesn't seem justified because the only known mechanism for Gcn1 to reduce ternary complex formation is via activation of Gcn2. Are they proposing that mGcn1 down-regulates ternary complex formation via DRG2? Is there evidence for this for the yeast proteins?

-p.13, regarding the statement: "As described above, p21 translation is increased by a decrease in the ternary complex. Indeed, the basal level of eIF2α phosphorylation appeared to decrease in both Gcn1l1ΔRWDBD and Gcn2 KO MEFs (Fig 4)." Unless I'm mistaken, the cited evidence showed that p21 translation is dependent on high-levels of the ternary complex; which seems at odds with the suggestion that increased p21 translation would result from decreased ternary complex formation owing to an elevated basal level of eIF2a phosphorylation in the ΔRWDBD mutant.

Response: Thank you for the comments and we agree with the reviewer's comment. We corrected the original sentence to "Although both OLA1 and GCN1 can mediate stress-induced decreases in ternary complex formation, the reasons for similarity is not clear at present and should be clarified in the future analysis". Furthermore, we corrected the sentence "p21 protein levels are increased by a decrease in ternary complex" to "p21 protein levels are increased by a decrease in ternary complex".
